# Supplementary material for: Dietary Methionine Deficiency Enhances Genetic Instability in Murine Immune Cells
Source: Int J Mol Sci. 2021 Feb 27;22(5):2378. doi: 10.3390/ijms22052378 (PMC7956689; doi:10.3390/ijms22052378)
Supplement: Supplementary file 1 [file ijms-22-02378-s001.pdf]

Supplementary Table 1. Nutritional characteristics of the methionine-adequate (TD.140520), methionine-deficient (TD.90262), and methionine-supplemented (TD. 160241) diets used in the study.

Formula	TD.140520 g/Kg	TD. 90262 g/kg	TD. 160241 g/kg
Sucrose	445.297	455.294	432.197
Corn Starch	198.783	200	198.783
Corn Oil	100	100	100
Cellulose	30	30	30
Mineral Mix, AIN-76 (170915)	35	35	35
Calcium Phosphate, dibasic	3	3	3
L-Alanine	3.5	3.5	3.5
L-Arginine HCl	12.1	12.1	12.1
L-Asparagine	6	6	6.0
L-Aspartic Acid	3.5	3.5	3.5
L-Cystine	3.5	3.5	3.5
L-Glutamic Acid	40	40	40.0
Glycine	23.3	23.3	23.3
L-Histidine HCl, monohydrate	4.5	4.5	4.5
L-Isoleucine	8.2	8.2	8.2
L-Leucine	11.1	11.1	11.1
L-Lysine HCl	18	18	18.0
L-Methionine	6.5	0	19.5
L-Phenylalanine	7.5	7.5	7.5
L-Proline	3.5	3.5	3.5
L-Serine	3.5	3.5	3.5
L-Threonine	8.2	8.2	8.2
L-Tryptophan	1.8	1.8	1.8
L-Tyrosine	5	5	5.0
L-Valine	8.2	8.2	8.2
Vitamin Mix, Teklad (40060)	10	5*	10
Ethoxyquin, antioxidant	0.02	0.02	0.02
% by weight			
Protein	15.3	14.6	18.4
CHO	63.3	64.3	61.9
Fat	10	10	10

*- Vitamin Mix, w/o choline, A, D, E; Vitamin E, DL-alpha tocopheryl acetate (500 IU/g; 0.242 g/kg); Vitamin A Palmitate (500,000 IU/g; 0.0396 g/kg); Vitamin D3, cholecalciferol (500,000 IU/g; 0.0044 g/kg).

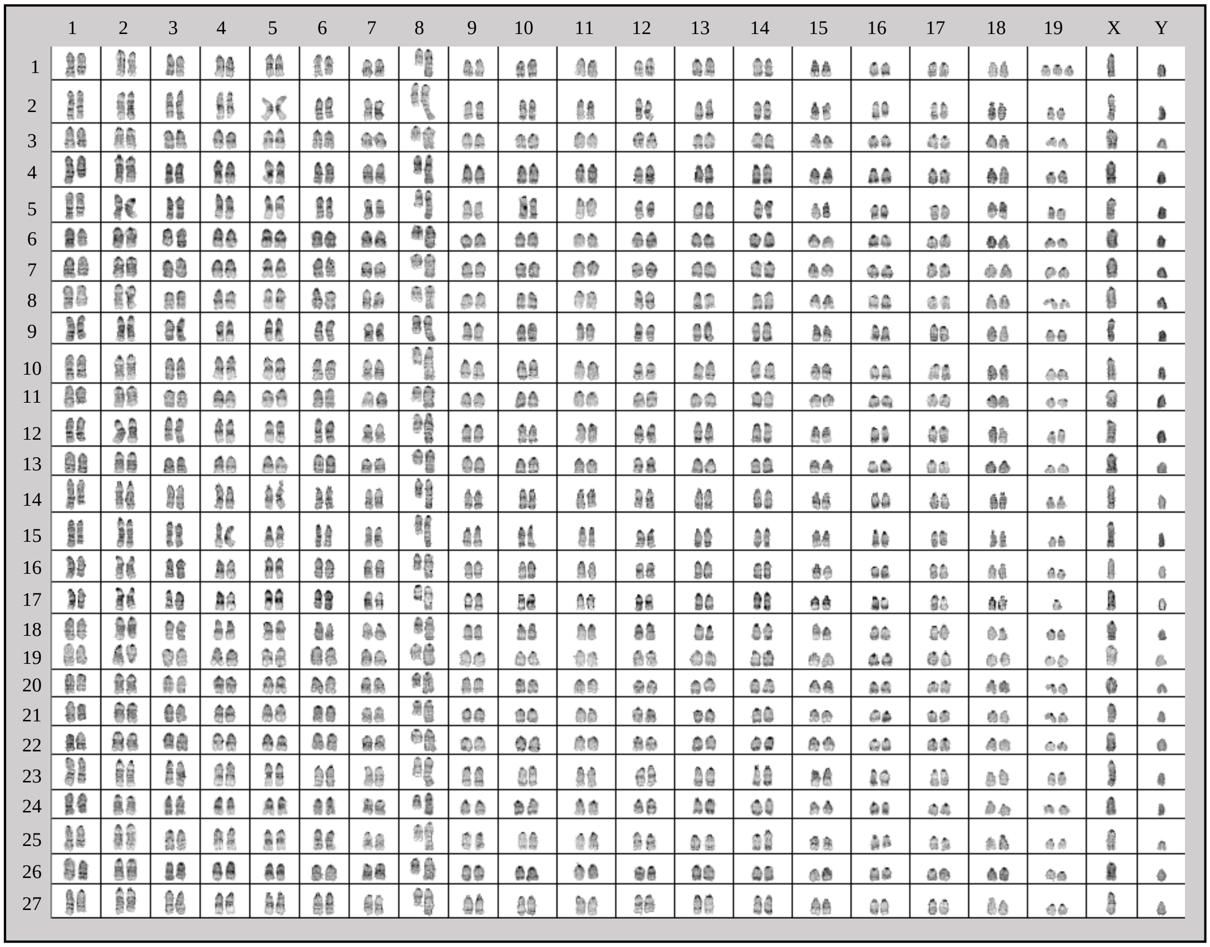

Supplemental Figure 1A: Clone 1 of RAW 264.7 cells growing in methionine adequate medium (1x) for 1 month.

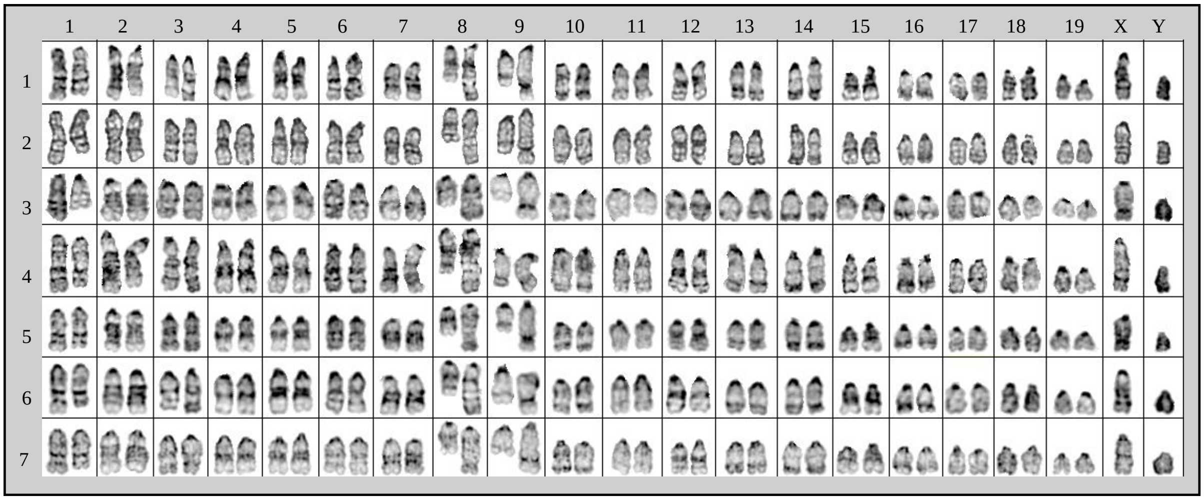

Supplemental Figure 1B: Clone 2 of RAW 264.7 cells growing in methionine adequate medium (1x) for 1 month.

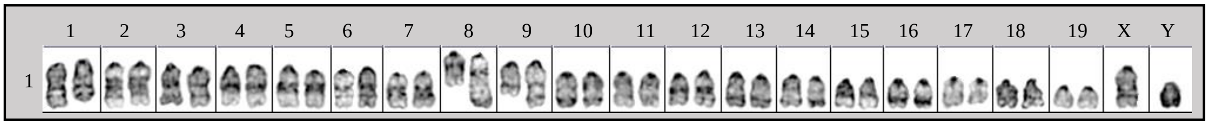

Supplemental Figure 1C: Clone 3 of RAW 264.7 cells growing in methionine adequate medium (1x) for 1 month.

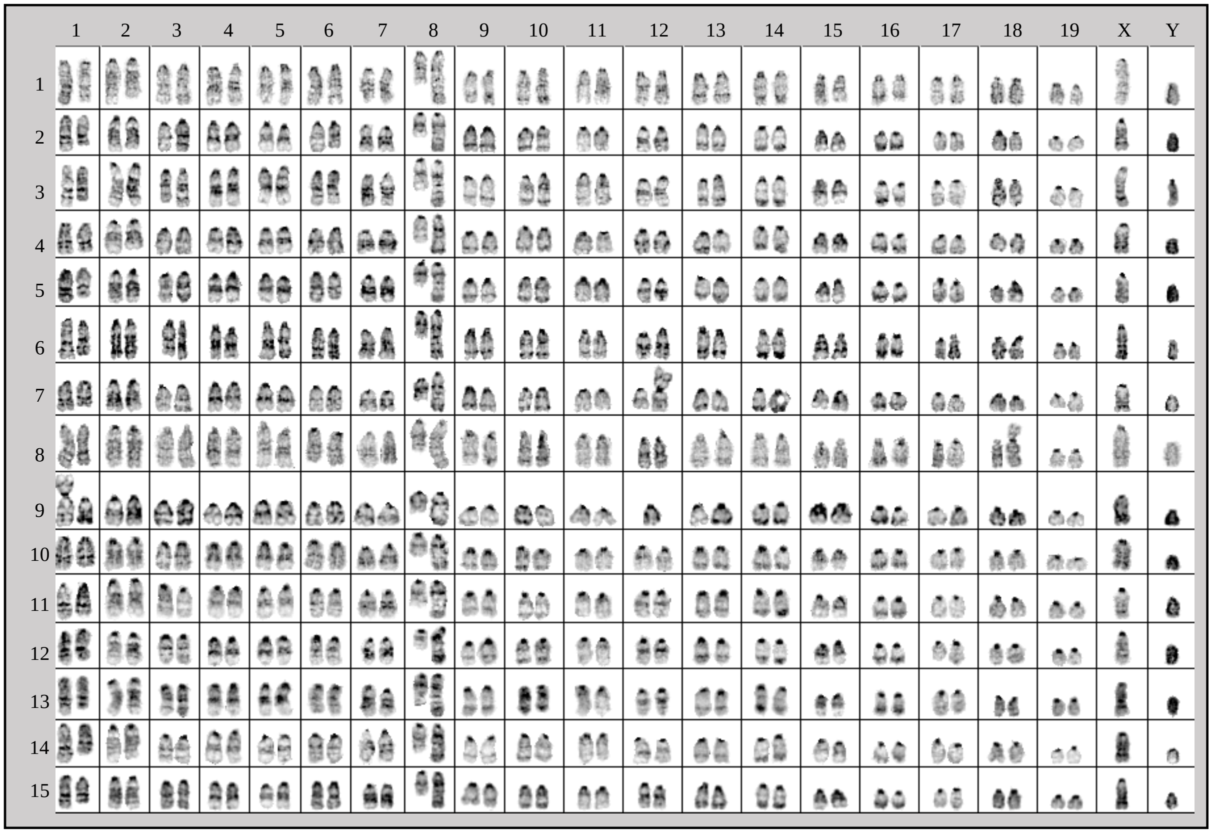

Supplemental Figure 2A: Clone 1 of RAW 264.7 cells growing in methionine deficient medium (0.25x) for 1 month.

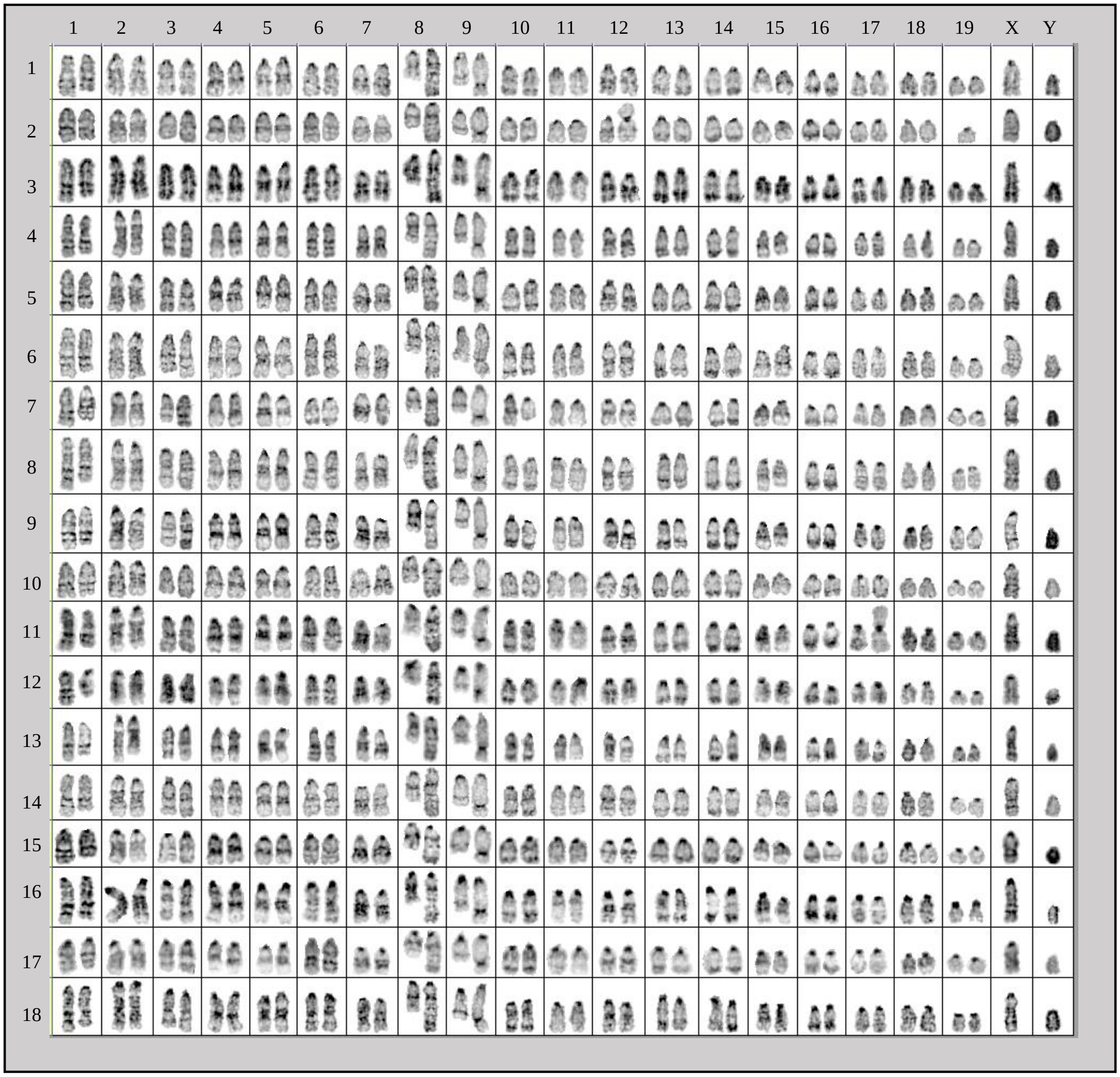

Supplemental Figure 2B: Clone 2 of RAW 264.7 cells growing in methionine deficient medium (0.25x) for 1 month.

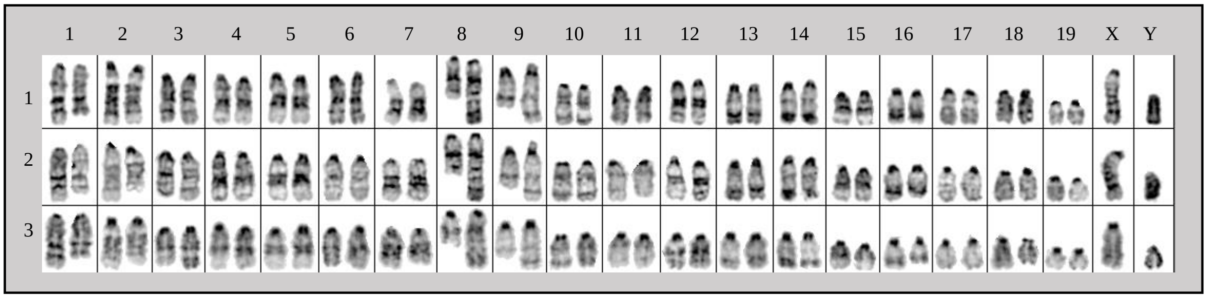

Supplemental Figure 2C: Clone 3 of RAW 264.7 cells growing in methionine deficient medium (0.25x) for 1 month.

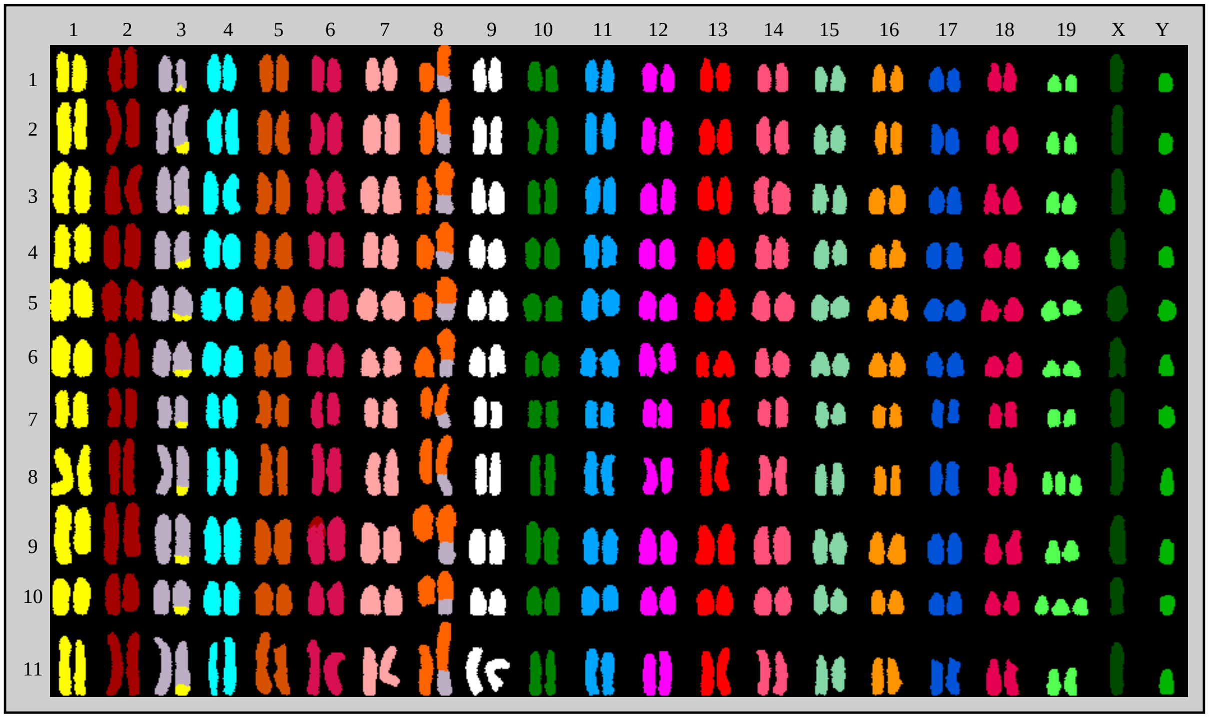

Supplemental Figure 3A: SKY analysis of RAW 264.7 cells growing in methionine adequate medium (1x) for 1 month.

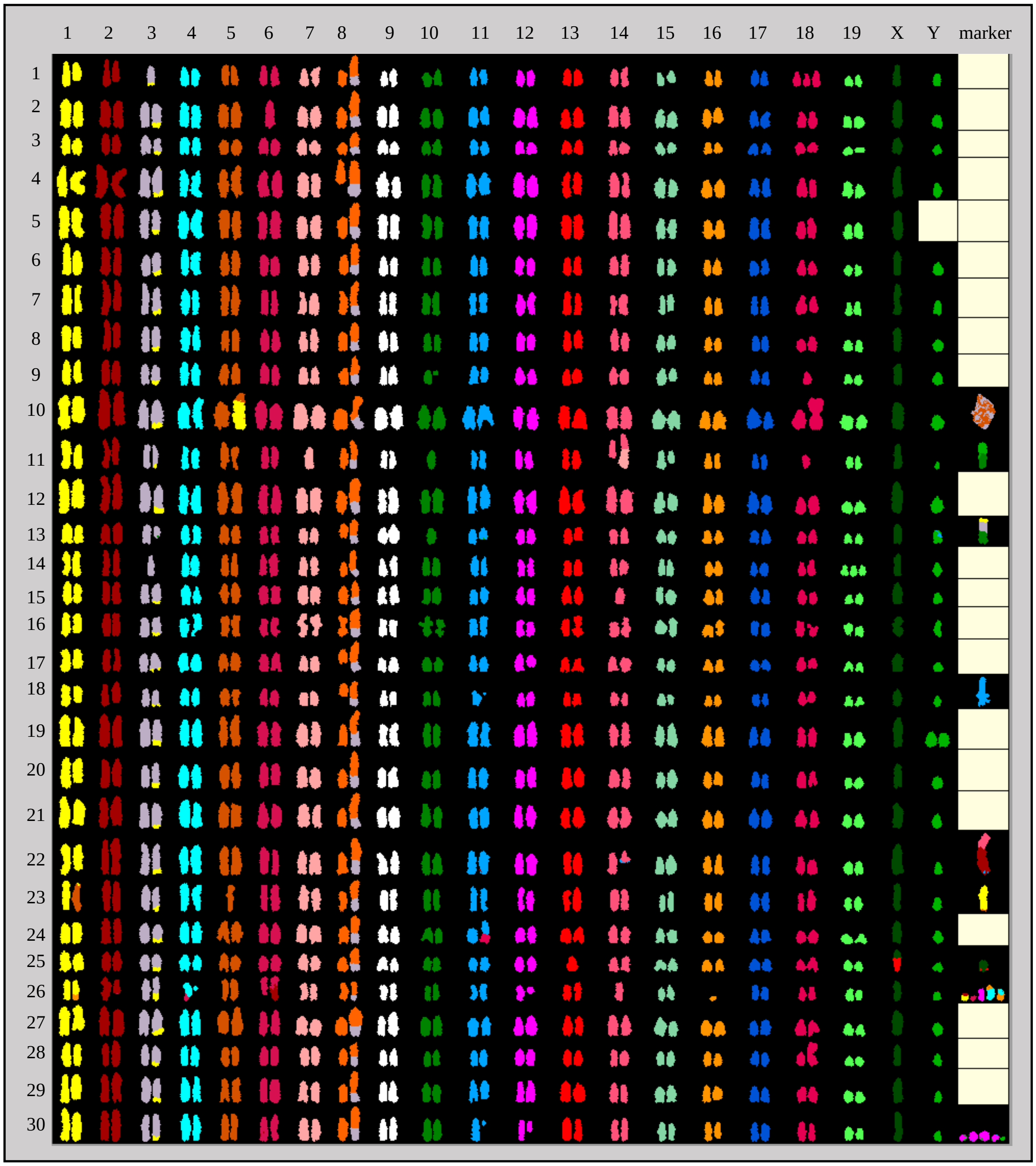

Supplemental Figure 3B: SKY analysis of Clone 1 of RAW 264.7 cells growing in methionine adequate medium (1x) for 1 month followed by 2 Gy of radiation.

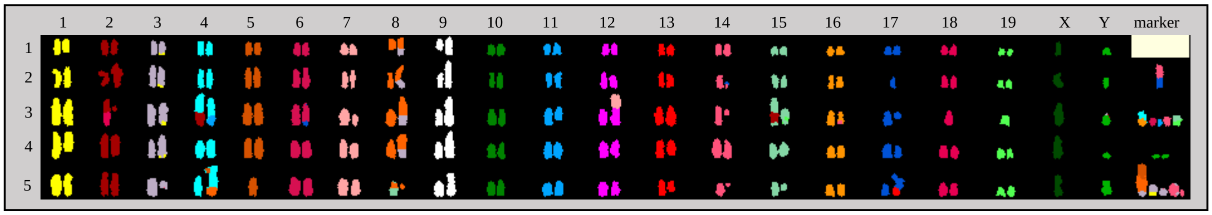

Supplemental Figure 3C: SKY analysis of Clone 2 of RAW 264.7 cells growing in methionine adequate medium (1x) for 1 month followed by 2 Gy of radiation.

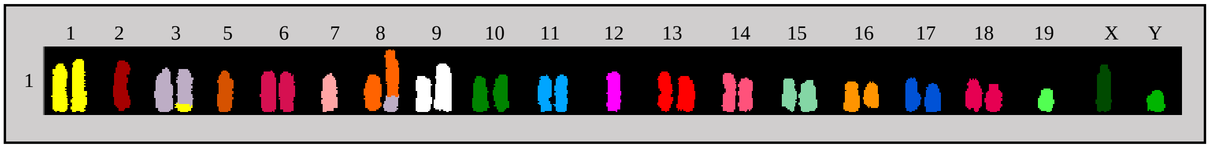

Supplemental Figure 3D: SKY analysis of Clone 3 of RAW 264.7 cells growing in methionine adequate medium (1x) for 1 month followed by 2 Gy of radiation.

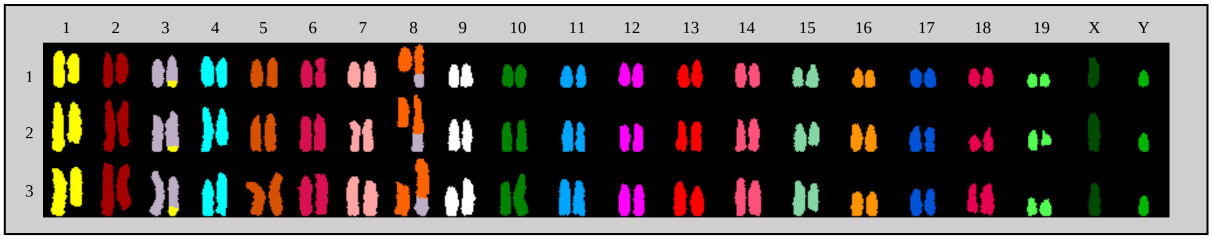

Supplemental Figure 4A: SKY analysis of Clone 1 of RAW 264.7 cells growing in methionine deficient medium (0.25x) for 1 month.

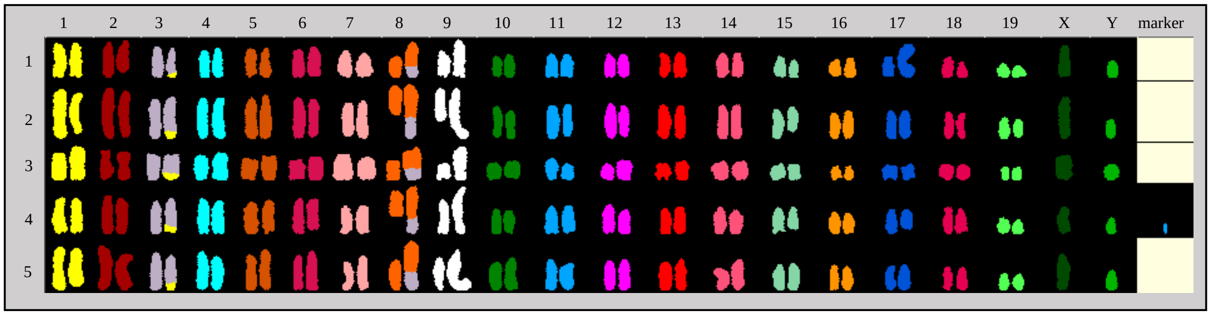

Supplemental Figure 4B: SKY analysis of Clone 2 of RAW 264.7 cells growing in methionine deficient medium (0.25x) for 1 month.

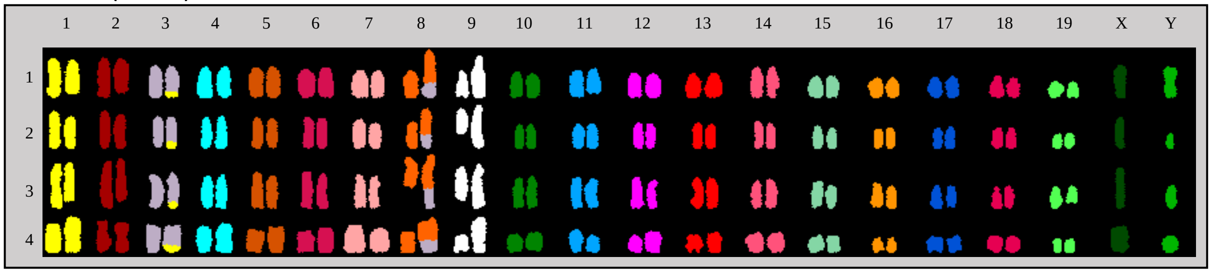

Supplemental Figure 4C: SKY analysis of Clone 3 of RAW 264.7 cells growing in methionine deficient medium (0.25x) for 1 month.

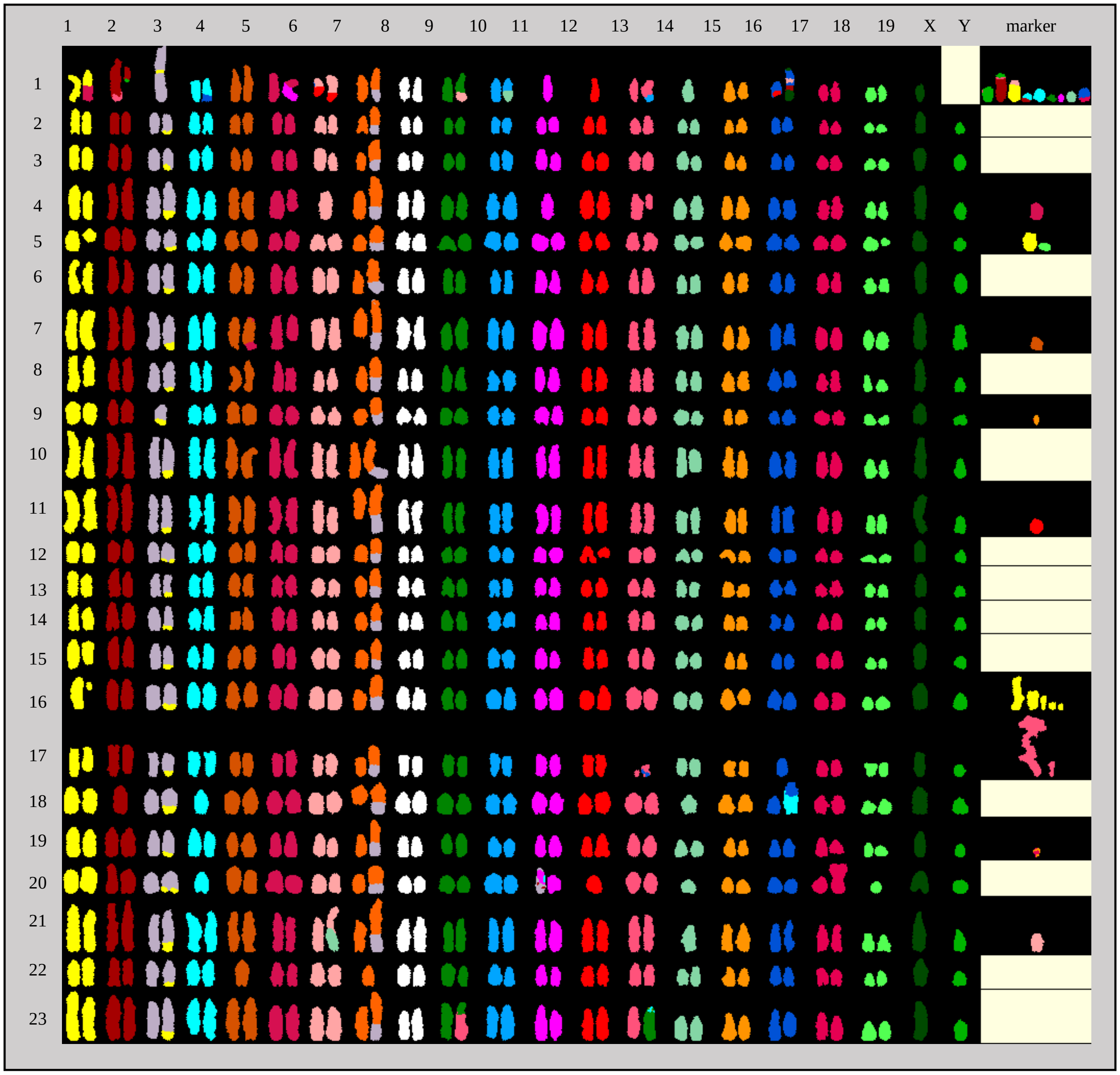

Supplemental Figure 4D: SKY analysis of Clone 1 of RAW 264.7 cells growing in methionine deficient medium (0.25x) for 1 month followed by 2 Gy of radiation.

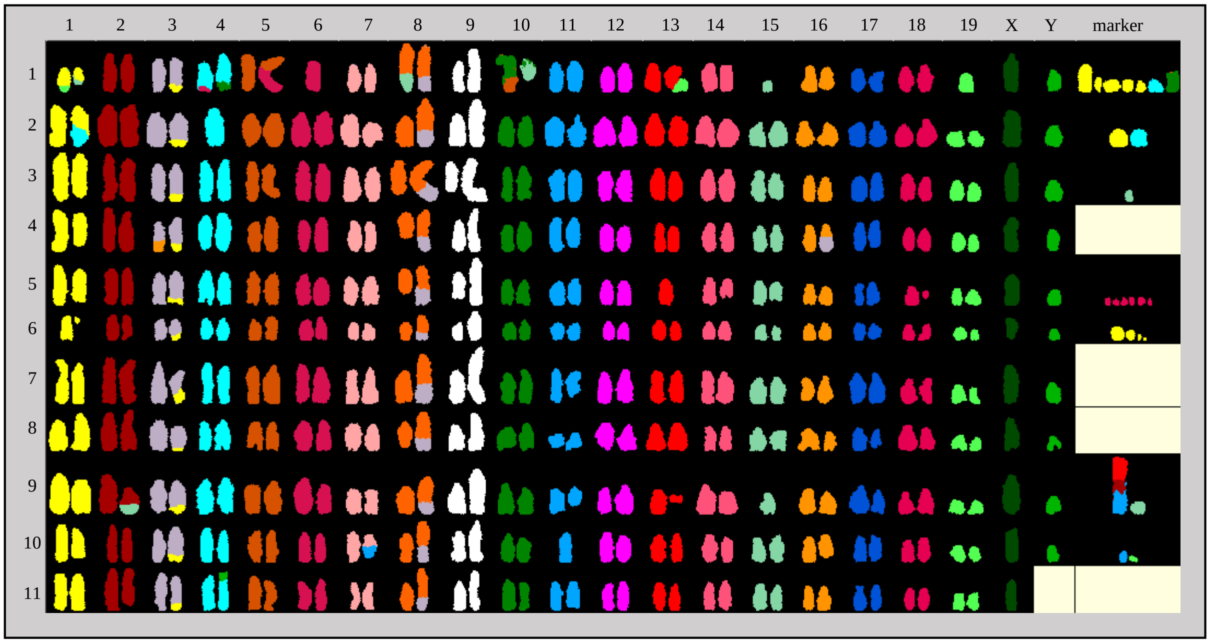

Supplemental Figure 4E: SKY analysis of Clone 2 of RAW 264.7 cells growing in methionine deficient medium (0.25x) for 1 month followed by 2 Gy of radiation.

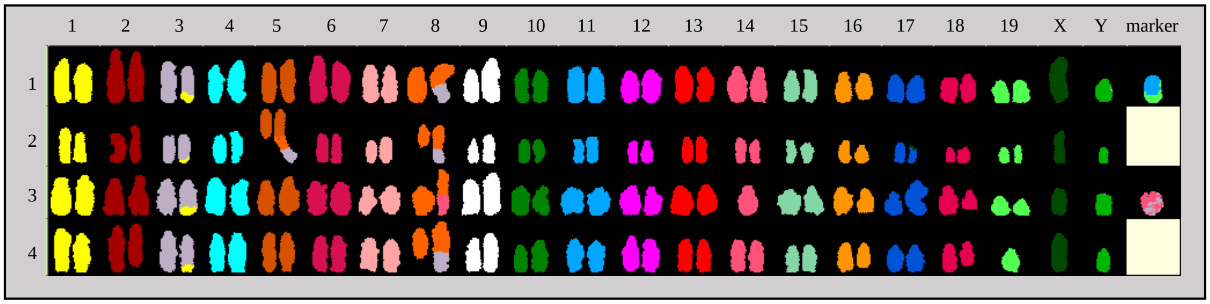

Supplemental Figure 4F: SKY analysis of Clone 3 of RAW 264.7 cells growing in methionine deficient medium (0.25x) for 1 month followed by 2 Gy of radiation.
